# The OXI1 Kinase Pathway Mediates *Piriformospora indica*-Induced Growth Promotion in Arabidopsis

**DOI:** 10.1371/journal.ppat.1002051

**Published:** 2011-05-19

**Authors:** Iris Camehl, Corinna Drzewiecki, Jyothilakshmi Vadassery, Bationa Shahollari, Irena Sherameti, Celine Forzani, Teun Munnik, Heribert Hirt, Ralf Oelmüller

**Affiliations:** 1 Friedrich Schiller University Jena, Institute of General Botany and Plant Physiology, Jena, Germany; 2 URGV Plant Genomics, INRA-CNRS-University of Evry, Evry, France; 3 Section Plant Physiology, Swammerdam Institute for Life Sciences, University of Amsterdam, Amsterdam, The Netherlands; 4 College of Science, King Saud University, Riyadh, Saudi Arabia; Massachusetts General Hospital, Harvard Medical School, United States of America

## Abstract

*Piriformospora indica* is an endophytic fungus that colonizes roots of many plant species and promotes growth and resistance to certain plant pathogens. Despite its potential use in agriculture, little is known on the molecular basis of this beneficial plant-fungal interaction. In a genetic screen for plants, which do not show a *P. indica-* induced growth response, we isolated an Arabidopsis mutant in the *OXI1* (*Oxidative Signal Inducible1)* gene. OXI1 has been characterized as a protein kinase which plays a role in pathogen response and is regulated by H_2_O_2_ and PDK1 (3-PHOSPHOINOSITIDE-DEPENDENT PROTEIN KINASE1). A genetic analysis showed that double mutants of the two closely related *PDK1.1* and *PDK1.2* genes are defective in the growth response to *P. indica*. While *OXI1* and *PDK1 gene* expression is upregulated in *P. indica*-colonized roots, defense genes are downregulated, indicating that the fungus suppresses plant defense reactions. PDK1 is activated by phosphatidic acid (PA) and *P. indica* triggers PA synthesis in Arabidopsis plants. Under beneficial co-cultivation conditions, H_2_O_2_ formation is even reduced by the fungus. Importantly, phospholipase D (PLD)α1 or PLDδ mutants, which are impaired in PA synthesis do not show growth promotion in response to fungal infection. These data establish that the *P. indica*-stimulated growth response is mediated by a pathway consisting of the PLD-PDK1-OXI1 cascade.

## Author Summary

Like many root-colonizing microbes, the primitive Basidiomycete fungus *Piriformospora indica* colonizes the roots of many plant species and promotes their growth. The lack of host specificity suggests that the plant response to this endopyhte is based on general signalling processes. In a genetic screen for Arabidopsis plants, which do not show a *P. indica-*induced growth response, we isolated a mutant in the *OXI1* (*Oxidative Signal Inducible1)* gene. Previously, this protein kinase has been shown to play a role in pathogen response and is regulated by H_2_O_2_ and PDK1 (3-PHOSPHOINOSITIDE-DEPENDENT PROTEIN KINASE1). A genetic analysis showed that deletion of PDK1 also abolishes the growth response to *P. indica*. PDK1 is activated by phosphatidic acid (PA). *P. indica* triggers PA synthesis and mutants impaired in PA synthesis do not show growth promotion in response to fungal infection. Since defense processes are repressed by *P. indica*, we propose that a pathway consisting of the PLD-PDK1-OXI1 cascade mediates the *P. indica*-induced growth response.

## Introduction

The endophytic fungus *Piriformospora indica*, a cultivable basidiomycete of Sebacinales, colonizes the roots of many plant species including Arabidopsis [1], [2]. Like other members of Sebacinales, *P. indica* is found worldwide in association with roots [3], and stimulates growth, biomass and seed production of the hosts [1], [2], [4]–[11]. The fungus promotes nitrate and phosphate uptake and metabolism [6], [12], [13]. *P. indica* also confers resistance against abiotic [7], [14], [15] and biotic stress [2], [16]. The broad host range of *P. indica* indicates that the beneficial interaction may be based on general recognition and signalling pathways. Little is yet understood about the molecular steps leading to *P. indica*-induced growth promotion. Plant growth can be induced by a fungal exudate component [9], suggesting the involvement of specific receptors at the plant cell surface. In support of this hypothesis, an atypical receptor kinase with leucine-rich repeats was identified to be required for the growth response in Arabidopsis [5]. Moreover, a rapid increase in the intracellular calcium concentration in the root cells indicates that an intracellular signalling cascade is triggered early upon plant-fungal interaction [9]. So far, however, no further components of the signalling pathway have been identified.

In mammals, the phospholipid-binding 3-PHOSPHOINOSITIDE-DEPENDENT PROTEIN KINASE1 (PDK1) sustains and regulates the balance between growth, cell division and apoptosis [17]–[19]. PDK1 is a member of the cAMP-dependent protein kinase A / protein kinase G / protein kinase C (AGC) kinase family [17] and the Arabidopsis homolog AtPDK1 is regulated by binding to the lipid phosphatidic acid (PA) [20], [21]. Phospholipase D (PLD)α1 is the main producer of PA in Arabidopsis roots [22]. In plants, PA is a second messenger [23], [24] that links lipid signalling to oxidative stress signalling [25], e.g. during abscisic acid-induced stomatal closure or defense against pathogens [26]–[28]. PDK1 is the only AGC kinase in plants with an identifiable lipid-binding domain [20], [21], [29], [30].

OXIDATIVE SIGNAL INDUCIBLE1 (OXI1) is a serine/threonine kinase necessary for oxidative burst-mediated signalling in *Arabidopsis* roots [20], [31]. OXI1 is a member of the AGC protein kinase family (also called AGC2-1 [30]) and its expression is induced by H_2_O_2_ [31]. OXI1 is required for full activation of the two mitogen-activating protein kinases 3 and 6 (MPK3 and MPK6) after treatment with reactive oxygen species (ROS) or elicitors and for different ROS-mediated processes including basal resistance to *Hyaloperonospora arabidopsidis* (previously known as *Peronospora parasitica*) infection and root hair growth [31]. Among all AGC kinases in Arabidopsis [30], AGC2-2 might be considered as an OXI1 homolog, however this kinase has not yet been investigated. The active OXI1 phosphorylates and thus activates the downstream serine/threonine kinase PTI1-2 in response to ROS and phospholipid signals [21], and many of these signals derive from microbial pathogens or elicitors, such as cell wall fragments or specific protein factors released by pathogens [32], [33]. Besides ROS, OXI1 is also activated by PDK1 [20].

In this work, we report on the results of a genetic screen for Arabidopsis mutants, which do not respond to *P. indica.* By positional cloning, we have identified *OXI1* as the responsible gene for the growth phenotype induced by *P. indica*. Since OXI1 is an AGC protein kinase that can be activated by H_2_O_2_ and PDK1, we also tested whether mutants in PDK1.1 and PDK1.2 are defective in the *P. indica*-induced growth phenotype. We found that *pdk1.1 pdk1.2* double knock out mutants do not respond to *P. indica*. The fungus stimulates PA, but not H_2_O_2_ synthesis in Arabidopsis plants. PA is produced by several pathways including by PLD. When PA synthesis was reduced by inactivation of phospholipase D (PLD)α1 or PLDδ, the *P. indica*-induced growth promotion was compromised. These results suggest that *P. indica* stimulates growth by PA-mediated activation of PDK1, which subsequently activates OXI1.

## Results

### Beneficial interaction between *P. indica* and Arabidopsis requires OXI1

**Figure 1:**
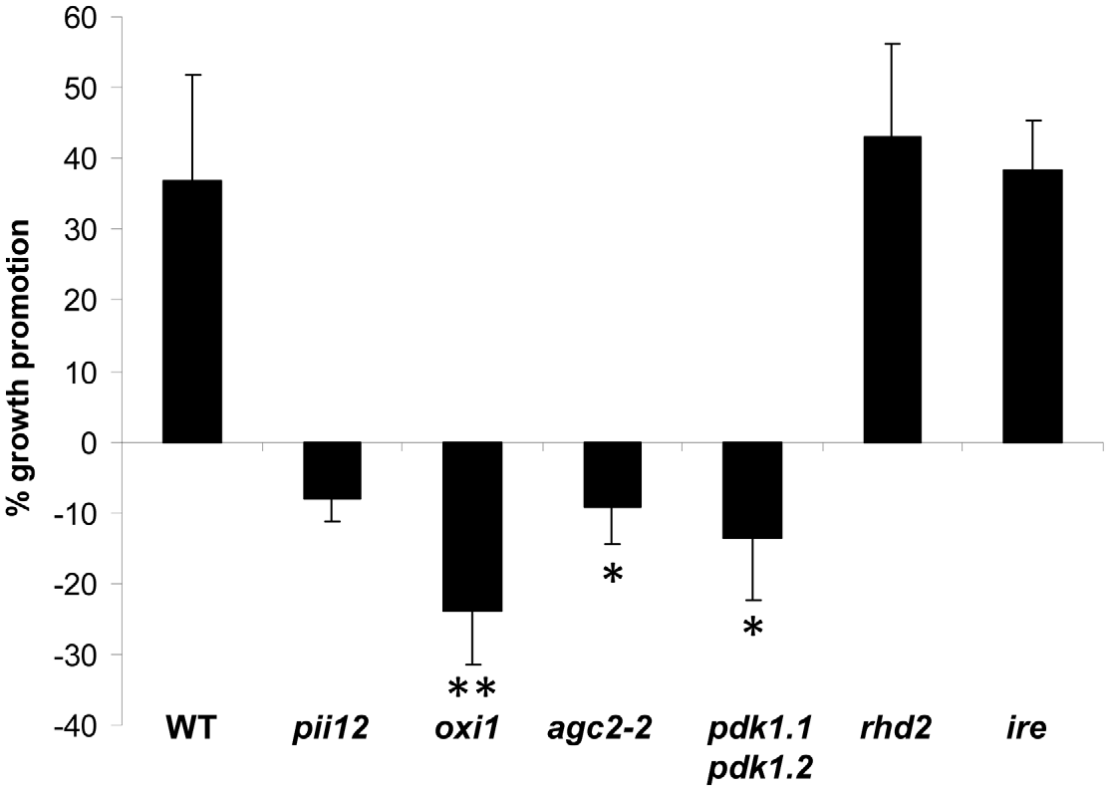
*P. indica*-mediated increase in fresh weight (%) of wild-type (WT) and mutant plants. Data are based on 5–9 independent experiments with 10 plants per treatment. Bars represent SEs (significant difference to WT; * p<0.05; ** p<0.001).

Arabidopsis plants co-cultivated with *P. indica* are taller than the uncolonized controls [1], [2]. On the basis of this growth phenotype, we searched for ethylmethane sulfonate-induced mutants, which grow like uncolonized plants or are smaller in the presence of the fungus. One of these mutants, called *Piriformospora indica*-*insensitive12* (*pii12*), was smaller in the presence of the fungus (Figure 1) and mapped to a region on chromosome 3 that included *oxi1*. Moreover, the *pii12* mutant had reduced root hair lengths and reduced *oxi1* mRNA levels in roots and shoots when compared to the wild-type (Figure S1 in Text S1). Sequence analysis uncovered that the mutant lacks a 19 bp segment upstream of the putative translation start site, while the coding region was intact. To clarify whether OXI1 is responsible for the absence of the *P. indica*-induced growth response in Arabidopsis, *pii12* was complemented with the full-length cDNA of *OXI1*. Three independent transformants had higher *OXI1* mRNA levels when compared to *pii12* and showed a growth response to the fungus, which was comparable to the wild type (Figure S2 in Text S1). An independent T-DNA insertion line for *oxi1* was used for further analysis, because it completely lacked *OXI1* mRNA (Figure S3A in Text S1). Like *pii12*, growth promotion by *P. indica wa*s inhibited in *oxi1* plants (Figure 1 and Figure S2 in Text S1). These results confirm that a deletion in the *OXI1* promoter region is responsible for the absence of the growth response of Arabidopsis plants to *P. indica.* We conclude that *P. indica*-induced growth promotion in Arabidopsis requires OXI1.

### H_2_O_2_ production is not stimulated upon fungal infection of Arabidopsis roots

Previously, it was shown that *OXI1* is induced by H_2_O_2_ in the roots [31]. However, H_2_O_2_ measurements and staining of colonized wild type roots with nitrobluetetrazolium chloride (NBT) uncovered that *P. indica* does not induce H_2_O_2_ accumulation [9]. Under growth promoting conditions, we even observed a repression of H_2_O_2_ accumulation in the roots (Figure S4C in Text S1). Also high concentrations of fungal hyphae, which are no longer beneficial for the plants, did not result in H_2_O_2_ production in the roots (H_2_O_2_ levels, no fungal treatment: 17.4±2.1 nmol/g fresh weight; non-beneficial interaction: 17.1±1.7 nmol/g fresh weight; n = 9 independent experiments).

### Root hair mutant *ire* and *rhd2* plants are not compromised in *P. indica*-induced growth promotion of Arabidopsis

The inability of *oxi1* plants to respond to *P. indica* might be caused by their shorter root hairs [31]. However, mRNA levels for the *P. indica* translation elongation factor1 (*Pitef1*) were comparable in *oxi1* and wild-type roots (Figure S5 in Text S1), indicating that root colonization does not differ from the wild-type in *oxi1*.

We also investigated the interaction of *P. indica* with two other mutants with reduced root hair phenotypes: the AGC kinase *ire* and the NADPH oxidase *rhd2* ([34], [35] Figure S3B in Text S1). Growth of these mutants was promoted by *P. indica* (Figure 1), and the degree of root colonization was again comparable to the wild-type (Figure S5 in Text S1). Therefore, the root hair phenotype does not seem to be responsible for the impaired interaction of *oxi1* with *P. indica*. Furthermore, among the *RHD* genes expressed in Arabidopsis roots, *RHD2* shows the highest expression level and RHD2 is responsible for most of the H_2_O_2_ production in the roots [35]. Thus, the lower H_2_O_2_ production in *rhd2* roots does not compromise the beneficial plant-fungal interaction.

### AGC2-2, a homolog of OXI1, is required for *P. indica*-induced growth promotion

AGC2-2 (At4g13000) is the closest homolog of OXI1 (see phylogenetic tree in [30]) and shares >60% sequence identity to OXI1. Both kinases contain an aspartic acid residue in their active site (D_149_ in OXI1 and D_146_ in AGC2-2) and share a conserved PDK1 binding site, the FxxF motif, at their C-terminal ends [20]. However, in contrast to the *OXI1* mRNA level, the *AGC2-2* mRNA level is not regulated by ROS (https://www.genevestigator.com). *agc2-2* plants did not show any visible phenotype, produced the same amount of seeds, and – in contrast to *oxi1* [31] - root hairs of *agc2-2* plants were not shorter than those of wild-type plants (Figure S1B in Text S1). However, despite the fact that root colonization was not affected by the *agc2-2* mutation (Figure S5 in Text S1), *agc2-2* plants were compromised in the growth response to the fungus (Figure 1). Thus, besides OXI1, the so far uncharacterized AGC2-2 is important for *P. indica*-mediated growth promotion in Arabidopsis. Attempts to generate homozygous *oxi1 agc2-2* double knock out lines failed: among 98 F2 plants obtained from crosses of the two mutants, all plants, which were homozygote for either *oxi1* or *agc2-2* were heterozygote for the other kinase gene. This suggests that both OXI1 and AGC2-2 might play a role in embryogenesis in Arabidopsis.

### PDK1 is required for *P. indica*-induced growth promotion

**Figure 2:**
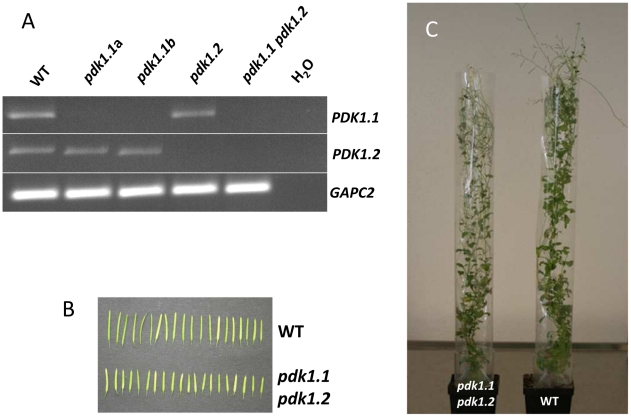
Characterization of PDK1 mutants. (**A**) *PDK1.1*, *PDK1.2* and *GAPC2* transcript amounts were determined by RT-PCR with gene-specific primer pairs. The *pdk1.1 pdk1.2* line does not contain *pdk1* transcripts. Two independent *pdk1.1* lines (a and b), *pdk1.2* and *pdk1.1 pdk1.2* were analysed. (**B**) Siliques and (**C**) phenotypes of *pdk1.1 pdk1.2* and wild-type plants are shown.

We next tried to identify the upstream components of the OXI1 cascade that is responsible for the fungal growth effect in plants. Previously, it was shown that PDK1 and H_2_O_2_ can activate OXI1 in Arabidopsis [20], [21]. Because *P. indica* infection did not alter H_2_O_2_ levels in Arabidopsis, we turned our attention to the two closely related *PDK1* genes, *PDK1.1* and *PDK1.2* (92% homology at the amino acid level), which are present in the Arabidopsis genome (cf. phylogenetic tree of AGC kinases in [30]). Both *PDK1* genes are expressed in roots. We generated a *pdk1.1 pdk1.2* double knock out line. RT-PCR analysis confirmed that neither *PDK1.1* nor *PDK1.2* transcripts can be detected in the double mutant line (Figure 2A). A phenotypic analysis revealed that *pdk1.1 pdk1.2* plants are smaller than the wild-type (Figure 2C), have shorter siliques (Figure 2B) and produce only 41%±6.8% (n = 23) of the seeds of the wild-type. Importantly, fungal induced growth promotion in *pdk1.1 pdk1.2 plants* was clearly compromised (Figure 1), whereas root colonization was comparable to the wild-type (Figure S5 in Text S1). Therefore, besides general functions in growth regulation, the combination of *PDK1.1* and *PDK1.2* is required for *P. indica*-induced growth promotion in Arabidopsis.

### PLDα1 and PLDδ are required for *P. indica*-mediated growth promotion

**Figure 3:**
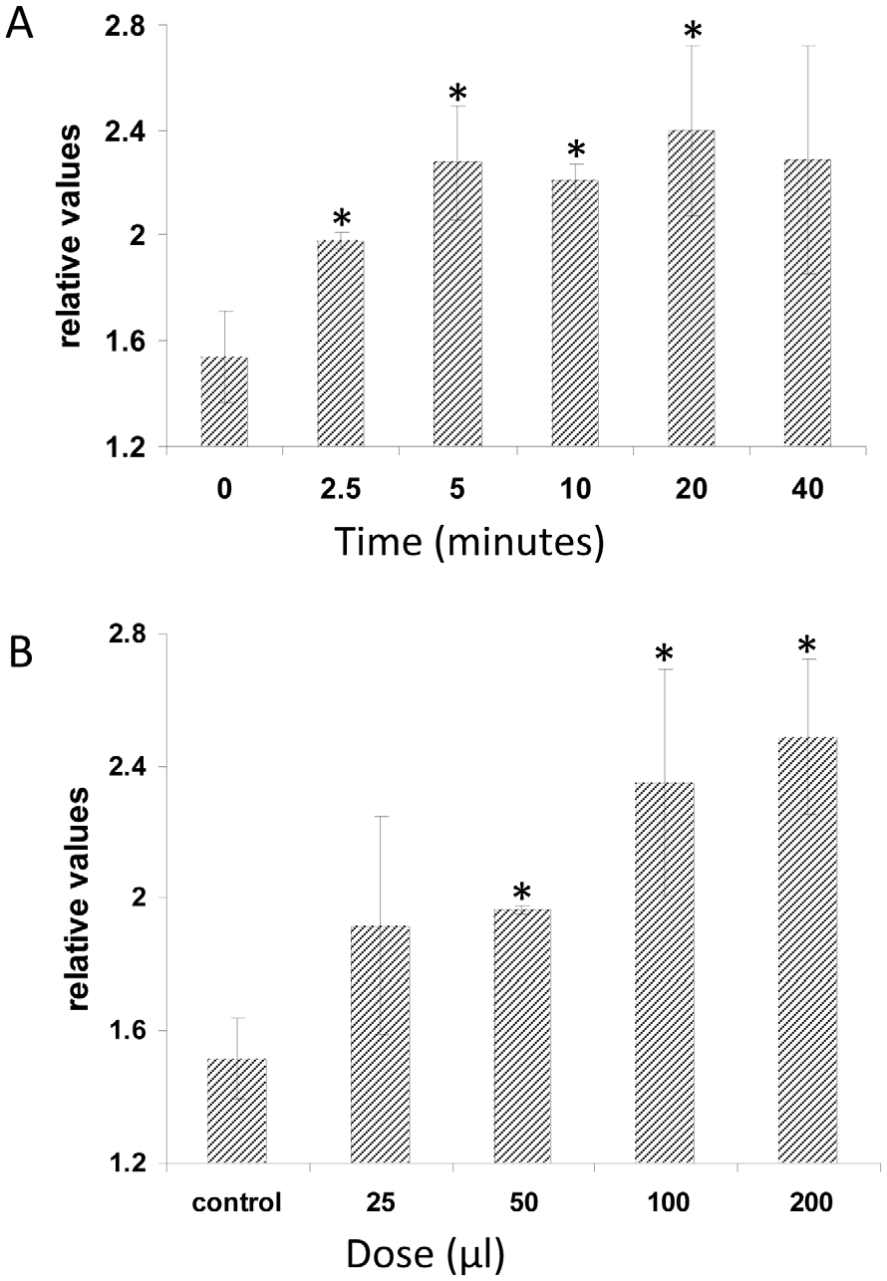
Plant PA levels increase in response to treatment with *P. indica* exudate. Five-days old seedlings were ^32^P_i_-labelled overnight and then treated with *P. indica* exudates. (A) Time series of plant PA amounts induced by 50 µl *P. indica* exudates. (B) Dose response curve of plant PA production in response to different amounts of *P. indica* exudate. Lipids were extracted, analysed by thin layer chromatography and PA levels were quantified by phosphoimaging. ^32^P-PA control levels were ∼1.5% of the total ^32^P-labelled lipids. The values represent: radioactivity [+*P. indica* extract/+buffer]. Bars represent SEs, based on 3 independent experiments. Bars marked with an asterisk are significantly different compared to wild type (p<0.05).

**Figure 4:**
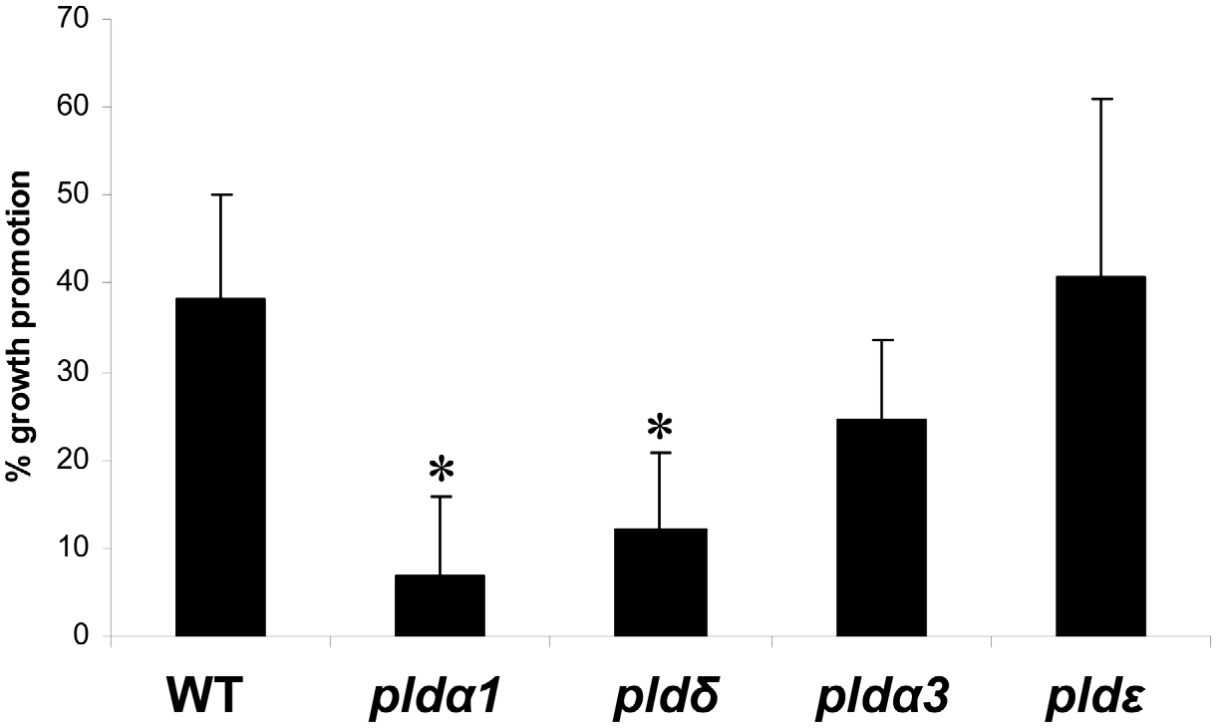
*P. indica*-induced increases in fresh weight (%) of wild-type and *pld* mutants. Data are based on at least three independent experiments with 10 plants per treatment. SEs are shown. Bars marked with an asterisk are significantly different compared to wild type (p<0.05).

After having established that PDK1 is an important component of the *P. indica*-induced growth response pathway, we tried to go even further up in the cascade to identify the regulator of the PDK1s. PDK1 in Arabidopsis is activated by PA. PA is synthesized by PLD and by PLC/diacylglycerol kinase. PA in roots is mainly generated by PLD activity [28], [36]. The Arabidopsis genome contains 12 genes for PLDs, which are classified into six types, PLDα (1–3), β (1 and 2), γ (1–3), δ, ε and ζ (1 and 2) [37]. The most abundantly expressed *pld* genes in roots are *pldα1* and *pldδ* [22], [38]. PLDα1 is responsible for most of the PA production in roots, and the PA content is severely reduced in the roots of *pldα1* knock out mutants [22]. Furthermore, wounding-induced PA production is completely eliminated in the *pldα1 pldδ* double knock out line [39]. Application of a *P. indica* exudate fraction, which promotes plant growth [9] stimulates PA accumulation in a time- and dose-dependent manner in the roots (Figure 3). Furthermore, the growth response of *pldα1* and *pldδ* insertion lines to *P. indica* was severely impaired (Figure 4). In comparison, the response of *pldα3* und *pldε* (Figure 4, Figure S3C in Text S1) plants to *P. indica* was similar to wild type. These results indicate that signals from the fungus activate PA synthesis via PLDα1 and PLDδ in the roots.

### Expression of defense-related genes is downregulated but independent of the OXI1 pathway under beneficial conditions

**Figure 5:**
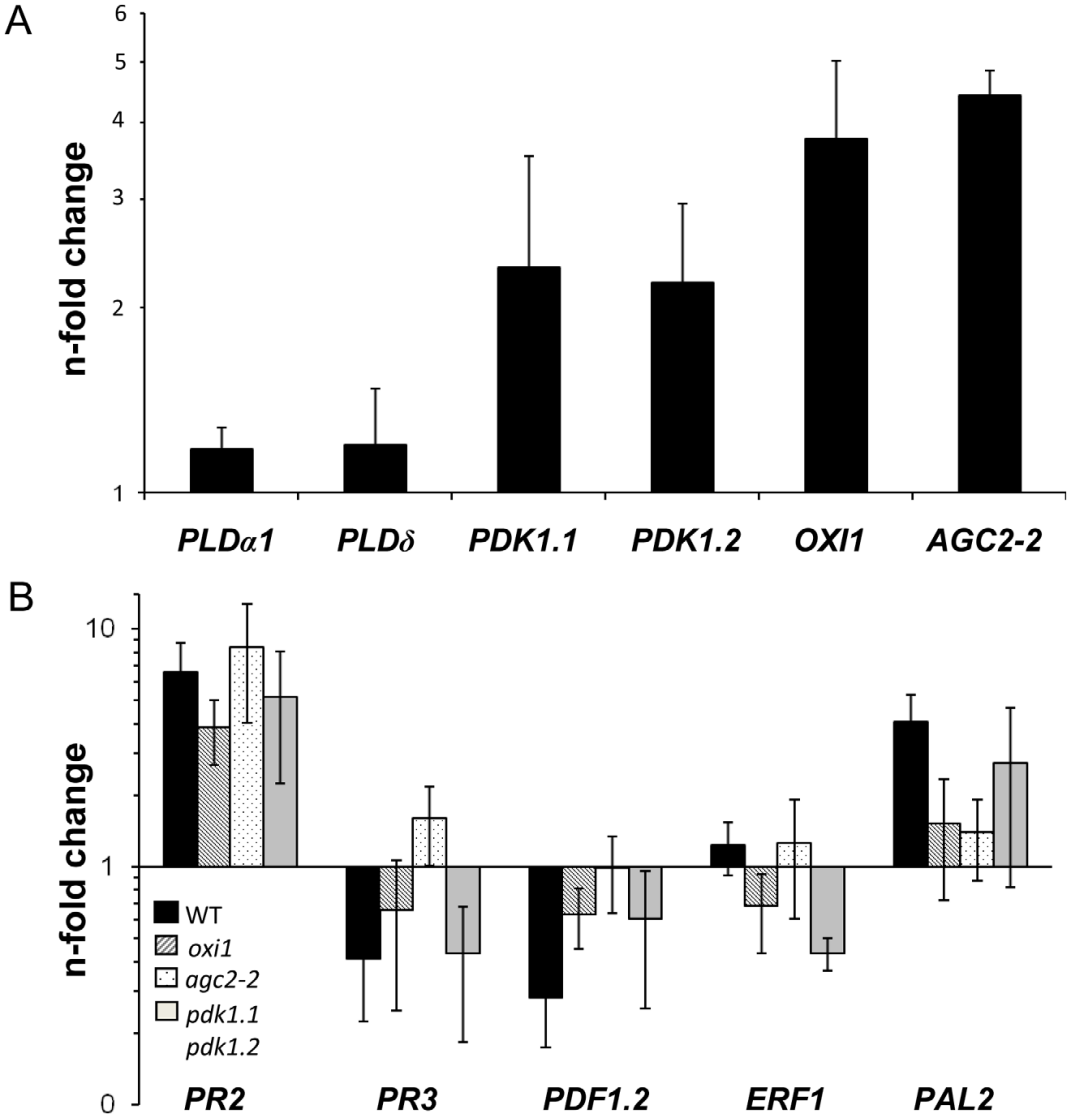
Expression levels of *PDK1*, *OXI1* and *AGC2-2* genes and defense genes in colonized wild-type or mutant roots relative to uncolonized control plants. Panel A shows *PDK1*, *OXI1* and *AGC2-2* expression levels and panel B shows expression levels of several defense genes. RNA was extracted from roots and real-time PCR analyses were performed with the housekeeping gene *UBQ5* as control. Calculations were performed according to [63]. Bars show the mean out of at least four independent experiments with SEs. Data are presented on a log scale.

Compared to uncolonized roots, the two *PDK1* mRNA levels were ∼2-fold higher and the *OXI1* and *AGC2-2* mRNA levels increased ∼4-fold in *P. indica*-colonized roots (Figure 5A). In contrast, three classical defense genes, which are targets of PDK1 and OXI1 signalling after pathogen infections (*PR3*, *PDF1.2*, *ERF1* [20], [21], [31]), are downregulated in *P. indica*-colonized wild-type roots (Figure 5B). Thus, upregulation of the *PDK1* and *OXI1* mRNA by *P. indica* does not result in the activation of the three defense genes. The expression level of defense genes is also downregulated in the colonized *pdk1.1 pdk1.2*, *oxi1* and *agc2-2* plants. *PR2* is mildly upregulated by the fungus, but this occurs also in the colonized mutants (Figure 5B). Thus, the regulation of the defense genes occurs independently of the OXI1 pathway under beneficial co-cultivation conditions of the two symbionts.

### Expression of defense-related genes is upregulated under non-beneficial conditions

**Figure 6:**
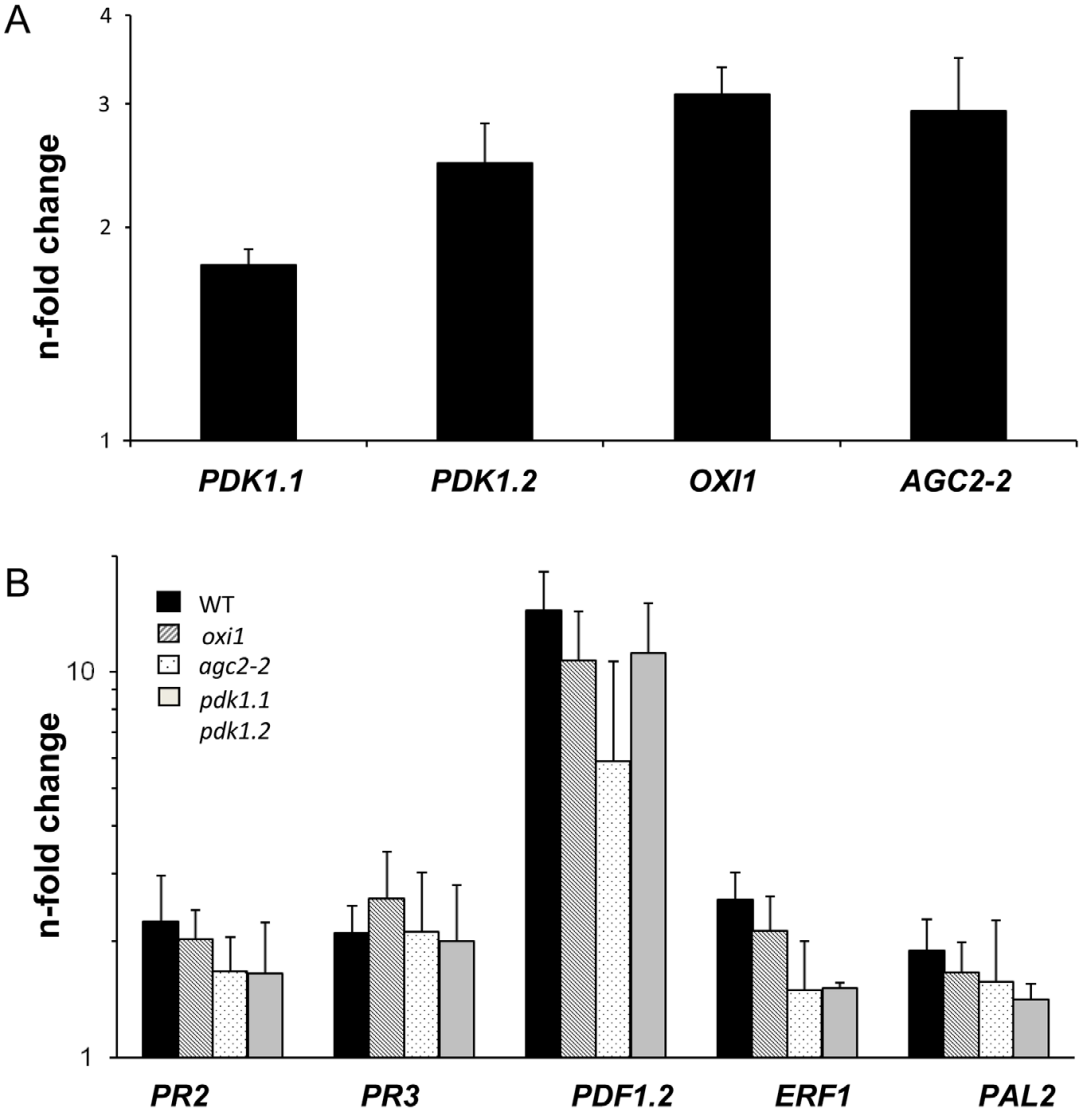
Expression levels of *PDK1*, *OXI1* and *AGC2-2* genes and defense genes after treatment with a high dosis of *P. indica* (for details, cf. Methods and Materials and Figure S4A,B in Text S1). Panel A shows *PDK1*, *OXI1* and *AGC2-2* expression levels and panel B shows expression levels of several defense genes. RNA was extracted from roots and real-time PCR analyses were performed with the housekeeping gene *GAPC2* as control. Calculations were performed according to [63]. Bars show fold-induction of RNA values from wild type and *agc* mutant roots 7 days after co-cultivation on a fungal lawn relative to the RNA levels from seedlings grown in the absence of the fungus. Bars show the mean out of at least three independent experiments with SEs. Data are presented on a log scale.

To test whether the PDK1, OXI1 and AGC2-2 kinases activate defense processes under non-beneficial conditions, we inoculated Arabidopsis plants with high doses of *P. indica.* Seven days after transfer to a dense fungal lawn, the seedlings still continued to grow (Figure S4A in Text S1), but visible accumulation of anthocyanin in the aerial parts were indicative of a stress response in the plants. No H_2_O_2_ accumulation would be detected under these co-cultivation conditions (Figure S4B in Text S1), however the *PDK1*, *OXI1* and *AGC2-2* mRNA levels were moderately upregulated (Figure 6A). In contrast to beneficial co-cultivation conditions, also defense genes, and in particular *PDF1.2*, were upregulated. However, this response was similar in wild type, *oxi1*, *agc2-2* and *pdk1.1 pdk1.2* mutants (Figure 6B). Therefore, upregulation of defense genes under non-physiological co-cultivation conditions is not mediated by the OXI1 pathway as well (Figure 6B).

## Discussion

**Figure 7:**
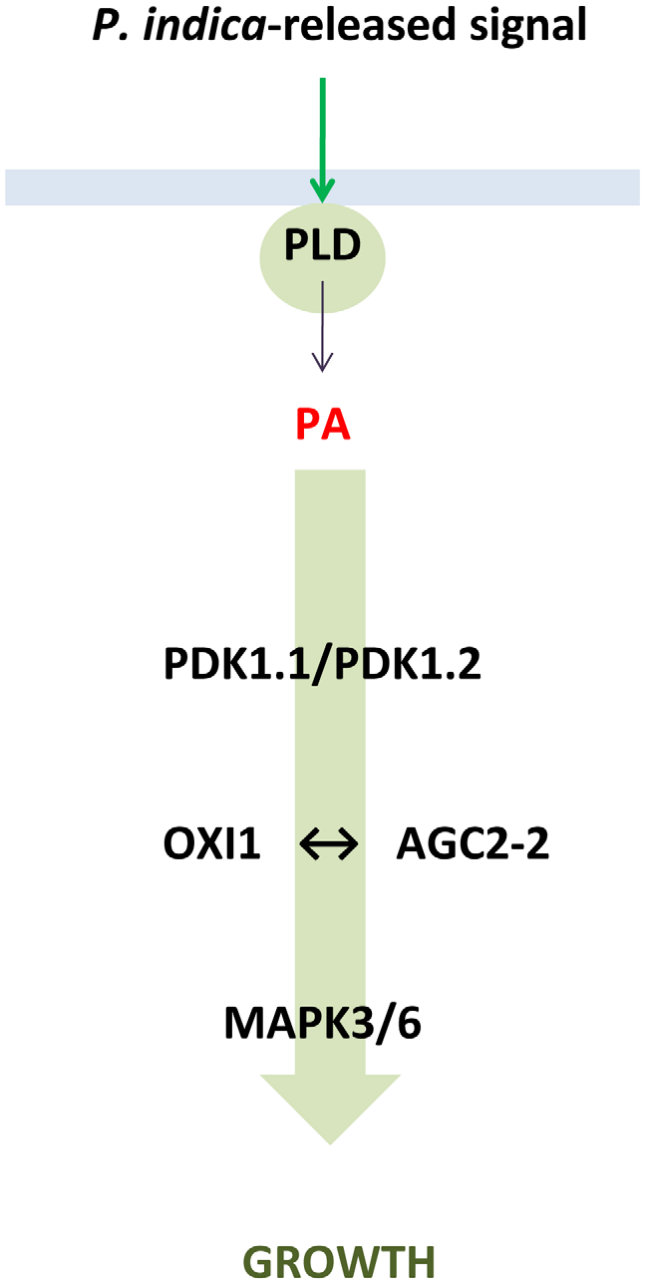
Proposed model describing the role of PLD, PA, AGC and MAP kinases in the beneficial interaction between *P. indica* and Arabidopsis. For MPK, see [9].

Growth promotion induced by *P. indica* in Arabidopsis depends on various compounds including phytohormones such as auxin and cytokinins [40], a balanced activation of defense responses in the roots [7], [41], the redox state in the cytoplasm [10] and sufficient nutrient supply [6]. In this work, we demonstrate that the OXI1 pathway is another important component, which mediates the beneficial interaction between *P. indica* and Arabidopsis. Moreover, we identified PLDα1, PLDδ and PDK1 as components, which are required for *P. indica*-induced growth promotion in Arabidopsis. Under beneficial co-cultivation conditions, *P. indica* stimulates PA synthesis, but not H_2_O_2_ production in Arabidopsis plants. The genetic evidence presented here and the biochemical data available for the OXI1 signalling pathway in pathogenic systems [20], [21], [28], [36] suggest that *P. indica* regulates plant growth via PA-stimulated PDK1 activation that subsequently triggers activation of the OXI1 and AGC2-2 protein kinases (Figure 7). The regulation of defense gene expression in response to nonbeneficial *P. indica* doses occurs also in *pdk1.1 pdk1.2*, *oxi1* and *agc2-2* mutants, indicating that the defense gene regulation is mediated by a pathway that functions independently of the OXI1 cascade.

### OXI1 and AGC2-2

The *pii12* and *oxi1* mutants are impaired in *P. indica*-induced growth promotion (Figure 1, Figure S2 in Text S1). The OXI1 kinase was shown to be induced by H_2_O_2_ and to activate defense responses against pathogen infections [20], [21], [31], [42]. However, H_2_O_2_ production is repressed in *P. indica*-colonized roots under beneficial co-cultivation conditions (Figure S4C in Text S1) and some defense genes are downregulated under beneficial conditions (Figure 5B). Exposure of Arabidopsis seedlings to high doses of the fungal hyphae induces a mild defense response, which occurs also in *oxi1* mutants (Figure 6B). Thus, OXI1 is required for the growth response but is not involved in defense gene activation in this beneficial interaction (cf. below). Interestingly, the *OXI1* overexpressor lines behaved like the wild-type (Figure S2 in Text S1) suggesting that wild-type amounts of the kinase are sufficient for the beneficial interaction. Furthermore, AGC2-2, a so far uncharacterized homolog of OXI1, is also required for the beneficial interaction. *AGC2-2* is not induced by H_2_O_2_, but by *P. indica* in wild-type roots (Figure 5A). Since attempts to isolate a homozygote *oxi1 agc2-2* double mutant failed and since the two single knock out lines fail to respond to *P. indica*, the two kinases have important and presumably different functions. Interestingly, this highly related pair of protein kinases resembles the OXI1-activated MAPKs MPK3 and MPK6, for which *MPK3* is inducible by pathogens, while *MPK6* is constitutively expressed and *mpk3 mpk6* double mutants are embryo-lethal [9], [43]. In mammalian systems, AGC kinases play important roles in growth and proliferation. The activation mechanism of AGC kinases from both kingdoms by lipids and their conserved epitopes [17] support the idea that OXI1 and AGC2-2 play a crucial role in regulating cell growth, division and/or elongation in response to the signals from *P. indica*.

Because *oxi1* mutants are also compromised in root hair growth, we tested two mutants with shorter root hairs, *ire* and *rhd2.* However, none of these mutants were impaired in the growth response to the fungus (Figure 1). Moreover, because *rhd2* is also impaired in full production of H_2_O_2_ in roots, the inability of *oxi1* to respond to *P. indica* is not caused by the reduced root hair phenotype or lower H_2_O_2_ levels in the roots.

### PDK1s, PLDs and PA

PA is an important second messenger and is involved in regulating plant growth, proliferation, biomass production, cell expansion, as well as responses to biotic and abiotic stresses [23], [24], [26]-[28], [36], [44]–[48]. In response to stresses, PA balances and fine-tunes the appropriate plant response to environmental signals [28], [36]. PA accumulation is induced by exudate preparations from *P. indica* in a dose- and time-dependent manner (Figure 3), suggesting that the roots sense signalling molecules released from the fungus. The requirement of the PA-activated PDK1s for the beneficial interaction suggests a participation in growth regulation, similar to mammalians [49]–[51]. Nitrate and phosphate uptake and metabolism is stimulated by *P. indica* and required for growth promotion [6], [12], [13]. PA also plays important roles in nitrogen [48], [52]–[54] and phosphate signalling [55], [56]. These results might provide a link between the *P. indica*-induced positive growth phenotype and the primary metabolism. Further experiments are necessary to investigate a role of PDK1, OXI1 and AGC2-2 in this respect.

Interestingly, in mammals and yeast, PDK1 is a central regulatory kinase, which phosphorylates and thus activates AGC kinases in response to rises in the levels of the second messenger phosphatidylinositol 3,4,5-trisphosphate [19], [57]. *pdk1* knock-out mice are embryo-lethal [58]. Since the Arabidopsis *pdk1.1 pdk1.2* double knock-out line is viable, activation of AGC kinases might be different in plant and mammalian systems [19], [57], [58].

PA is synthesized by PLD or phospholipase C/diacylgycerol kinase (PLC/DAG) [36]. PLDα1 and PLDδ are abundantly expressed in roots. We observed that their inactivation severely reduces *P. indica*-induced growth promotion (Figure 4). *pldα1* was shown previously to contain lower PA levels in the roots [22], has reduced wounding-induced PA production, and this response is completely eliminated in the *pldα1 pldδ* double knock out line [39]. PLDα1 and PA have also been implicated in regulating NADPH oxidase activity and the production of H_2_O_2_ in ABA-mediated stomatal closure [25]. The plasma-membrane-bound PLDδ is activated in response to H_2_O_2_ [59]. However, since H_2_O_2_ is not accumulating in response to *P. indica*, the lipases might have a different function and are differently regulated in this beneficial interaction. *PLDα1* and *PLDδ* expression is not induced by *P. indica*. PLDα1 activity is regulated by dynamic changes in intracellular Ca^2+^ levels (cf. [28]), and the Ca^2+^ levels in the root cytoplasm increases even faster in response to the same exudate fraction from *P. indica* that induces PA accumulation (Figure 3; [9]). These results suggest that signals from *P. indica* are decoded via the two intracellular second messengers PA and Ca^2+^. It remains to be determined how PA and Ca^2+^ cooperate to induce the appropriate plant responses, and which mechanisms determine whether they activate responses leading to a beneficial interaction or defense activation.

In conclusion, we demonstrate that in the beneficial interaction between *P. indica* and Arabidopsis the OXI1 pathway constitutes a protein kinase signalling pathway that confers growth stimulation (Figure 7). We propose a model whereby roots sense signals derived from *P. indica* by activating a signalling pathway that results in PA-mediated activation of PDK1, which subsequently activates the OXI1 and AGC2-2 protein kinases. Since MPK6 is a downstream target of OXI1 [31] and required for *P. indica*-mediated growth promotion [9], it is possible that MPK6 might be an additional component of this pathway. Future studies on the targets of the OXI1 pathway should help to clarify by which mechanism growth promotion occurs in plants and how this knowledge could be used to improve yield and productivity in agriculture. It also remains to be determined whether promotion of plant growth by mycorrhizal fungi or plant-growth promoting bacteria requires the same pathway, and how the Arabidopsis mutants analysed in this study respond to pathogens.

## Materials and Methods

### Growth conditions of plants and fungi, co-cultivation experiments

Wild-type *Arabidopsis thaliana* seeds and seeds from the homozygote T-DNA insertion lines were surface-sterilized and placed on Petri dishes containing MS nutrient medium [60]. After cold treatment at 4°C for 48 h, plates were incubated for 7 days at 22°C under continuous illumination (100 µmol m^−2^ sec^−1^). *P. indica* was cultured as described previously [1], [4] on Kaefer medium. Nine day-old *A. thaliana* seedlings were transferred to nylon disks (mesh size 70 µm) and placed on top of a modified PNM culture medium (5 mM KNO_3_, 2 mM MgSO_4_, 2 mM Ca(NO_3_)_2_, 0.01 µM FeSO_4_, 70 µM H_3_BO_3_, 14 µM MnCl_2_, 0.5 µM CuSO_4_, 1 µM ZnSO_4_, 0,2 µM Na_2_MoO_4_, 0.01 µM CoCl_2_, 10.5 g L^−1^ agar, pH 5.6), in 90 mm Petri dishes. Fungal plugs of 5 mm in diameter were placed at a distance of 1 cm from the roots. Control seedlings remain untreated. Plates were incubated at 22°C under continuous illumination from the side (80 µmol m^−2^ sec^−1^).

The following homozygote T-DNA insertion lines were used: *rhd2* (At5g51060; [35] obtained from Prof. V. Zársky, Prague, Czech Republic), *ire* (At5g62310) Salk_043276, *oxi1* (At3g25250) Gabi_355H08, *agc2-2* (At4g13000) Salk_083220, *pdk1.1a* (At5g04510) Salk_113251, *pdk1.1b* (At5g04510) Salk_007800, *pdk1.2* (At3g10540) Sail_450_B01, *pldα1-1* (At3g15730, [61]) Salk_067533, *pldδ* (At4g35790, [61]) Salk_023247, *pldα3* (At5g25370) Salk_122059, *pldε* (At1g55180) Koncz68434. *pdk1.1 pdk1.2* was generated by crosses between *pdk1.1* and *pdk1.2*.

### Experiments on vermiculite

6 week-old adult plants were used for interaction studies with *P. indica*. Arabidopsis seedlings, grown for 14 days on MS media, were transferred to vermiculite (rather than soil), because this allowed to harvest the intact roots including the lateral roots. The growth response of the plants to *P. indica* on soil and on vermiculite is comparable (data not shown). The vermiculite was mixed with the fungus (1%, w/v) which was dissolved in PNM medium. 70 ml of liquid PNM medium or inoculated PNM medium was used per plant. The fungal mycelium was obtained from two weeks old liquid cultures after the medium was removed and the mycelium was washed with an excess of distilled water. Cultivation occurred in pots in a temperature-controlled growth chamber at 22°C under short-day conditions (light intensity: 80 µmol m^−2^ sec^−1^). The sizes of the plants were monitored weekly and after six weeks the fresh weights of the shoots were determined and the roots harvested for RNA or DNA extraction.

### Experiments with the fungal lawn

12-day-old seedlings were directly transferred from MS medium to a plate with a fungal lawn. The fungal lawn was obtained by placing a fungal plug on Kaefer medium and the fungus was allowed to grow for 14 days at 24°C in the dark, before the seedlings were transferred to the plate. Control seedlings were transferred to Kaefer medium without the fungus. The plates were incubated for 7 days at 22°C under continuous illumination (80 µmol m^−2^ sec^−1^) from above. Fresh weights were determined (data not shown) and RNA was extracted of the root material.

### RNA analysis

RNA was isolated from the roots with an RNA isolation kit (RNeasy, Qiagen, Hilden, Germany). For quantitative RT-PCR, RNA from Arabidopsis roots grown in the absence or presence of *P. indica* was used. Reverse transcription of 1 µg of total RNA was performed with oligodT Primer. First strand synthesis was performed with a kit from Qiagen (Omniscript, Qiagen, Hilden, Germany). RT-PCR was conducted with the primer pairs given in Figure S6 in Text S1. *P. indica* was monitored with a primer pair for the translation elongation factor 1 (*Pitef1*; [62]). The colonized (and control) roots were removed from vermiculite, rinsed 6 times with an excess of sterile water and were frozen in liquid nitrogen for RNA or DNA extraction. One of the two plant genes (*GAPC2* and *UBQ5*) was used as housekeeping genes for Arabiopsis roots.

Semiquantitative analysis was performed after 27 PCR cycles: the products were analysed on 2% agarose gels, stained with ethidium bromide, and visualized bands were quantified with the ImageQuant 5.0 (GE Healthcare Life Sciences). Real-time quantitative RT-PCR was performed using the iCycler iQ real-time PCR detection system and iCycler software version 2.2 (Bio-Rad, Munich, Germany). For the amplification of the PCR products, iQ SYBR Supermix (Bio-Rad) was used according to the manufactureŕs instructions in a final volume of 23 µl. The iCycler was programmed to 95°C 2 min, 35× (95°C 30 s, 55°C 40 s, 72°C 45 s), 72°C 10 min followed by a melting curve programme (55–95°C in increasing steps of 0.5°C). All reactions were repeated twice. The mRNA levels for each cDNA probe were normalized with respect to the *GAPC2* and *UBQ5* message levels. Fold induction values were calculated with the ΔΔCP equation of Pfaffl (2001) [63]. The ratio of a target gene was calculated in the treated sample versus the untreated control in comparison to a reference gene. The primer pairs are given in Figure S6 in Text S1.

### H_2_O_2_ measurements

H_2_O_2_ was determined by an assay coupled to the peroxidase [64]. Roots (0,1 g) were homogenized in 1 mL 1 M HClO_4_/insoluble PVP (5%). The supernatant was clarified by centrifugation, adjusted to pH 5.6 with 5 M K_2_CO_3_ solution and incubated with 1U ascorbate oxidase for 10 min to oxidize the ascorbate. The reaction in 0.1 M phosphate buffer (pH 6.5), 3.3 mM 3-(dimethylamino) benzoic acid, 0.07 mM 3-methyl-2-benzothiazoline hydrazone and 0.3 U peroxidase was started by adding the oxidized extracts and followed by absorbance change at 590 nm and 25°C. NBT staining has been described previously [9].

### PA measurements

Arabidopsis seedlings (5-days-old) were labeled overnight in 400 µL buffer (2.5 mM MES-KOH, 1 mM KCl, pH 5.7) containing 10 µCi of carrier-free PO_4_^3−^. Samples (3 seedlings each) were treated by adding 100 µL water with or without elicitor for the times and concentrations indicated. Treatments were stopped by adding 50 µL 50% perchloric acid (w/v) and shaking the samples vigorously for 5 min. Liquid was then removed and replaced by 375 µL of CHCl_3_/MeOH/HCl [50∶100∶1 (v/v)] followed by 100 µL 0.9 % NaCl (w/v), to extract the lipids while shaking (10 min). A two-phase system was induced by the addition of 375 µL of CHCl_3_ and 200 µL of 0.9% (w/v) NaCl. The remainder of the extraction was performed as described before [32]. For quantitative analysis, lipids were separated by thin-layer chromatography (TLC) using heat-activated, potassium oxalate/EDTA-impregnated, silica TLC plates (Merck, 20×20×0.1 cm) and an alkaline solvent system of CHCl_3_/MeOH/25%NH_4_OH/H_2_O [90∶70∶4∶16 (v/v)], essentially as described in [65]. Phospholipids were visualized and quantified by phosphoimaging (Molecular Dynamics, Sunnyvale, CA, USA).

### Statistics

All data were analysed with one-side, unpaired students *t*-Test (p≤0.05) in Excel.

### Accession numbers

OXI1 (other names: AGC2; AGC2-1; OXIDATIVE SIGNAL-INDUCIBLE1; ATOXI1; MJL12.22), At3g25250, NP_189162.1; AGC2-2 (other names: F25G13.90; F25G13_90), At4g13000, NP_193036.1; PDK1.1 (other names: 3'-PHOSPHOINOSITIDE-DEPENDENT PROTEIN KINASE 1; ATPDK1; PDK1; T32M21.110), At5g04510, NP_568138.1; PDK1.2 (other names: PDK2; F13M14.18), At3g10540, NP_187665.2; RHD2 (other names: A. THALIANA RESPIRATORY BURST OXIDASE HOMOLOG C; ATRBOHC; K3K7.25; RBOHC; ROOT HAIR DEFECTIVE 2), At5g51060, NP_199919.1; IRE (other names: INCOMPLETE ROOT HAIR ELONGATION), At5g62310, NP_201037.1; PLDα1 (other names: MSJ11.13; PHOSPHOLIPASE D ALPHA 1; PLD), At3g15730, NP_188194.1; PLDδ (other names: ARABIDOPSIS THALIANA PHOSPHOLIPASE D DELTA; ATPLDDELTA; F4B14.60; PLDDELTA), At4g35790, NP_849501.1; PLDα3 (other names: F18G18.110; PHOSPHOLIPASE D ALPHA 3; PLDALPHA3), At5g25370, NP_197919.1; PLDε (other names: F7A10.25; PHOSPHOLIPASE D ALPHA 4; PLDALPHA4; PLDEPSILON), At1g55180, NP_175914.1

Information from http://www.ncbi.nlm.nih.gov/ and http://www.arabidopsis.org/

## Supporting Information

Text S1 Supporting information.
(DOC)
