# Supplementary material for: The OXI1 Kinase Pathway Mediates Piriformospora indica-Induced Growth Promotion in Arabidopsis
Source: PLoS Pathog. 2011 May 19;7(5):e1002051. doi: 10.1371/journal.ppat.1002051 (PMC3098243; doi:10.1371/journal.ppat.1002051)
Supplement: Text S1 — Supporting information. (DOC) [file ppat.1002051.s001.doc]

**Text S1**

**Figure S1**

**Characterization of *pii12* and *oxi1* mutants.**

1. *Piriformospora indica*-*insensitive12* (*pii12*) and *oxi1* mutant plants were analysed for *OXI1* mRNA levels in roots and leaves. The gel shows RT-PCR products for *OXI1* and *GAPC2* (control) of RNA from roots and leaves of 6 weeks-old wild-type (WT), *pii12* (EMS mutant) and *oxi1* (T-DNA insertion line) plants. The numbers represent relative *OXI1* mRNA levels based on real-time PCR analysis, whereas WT values are 100 and the *pii12* values are expressed relative to them (n= 6 plants).

**
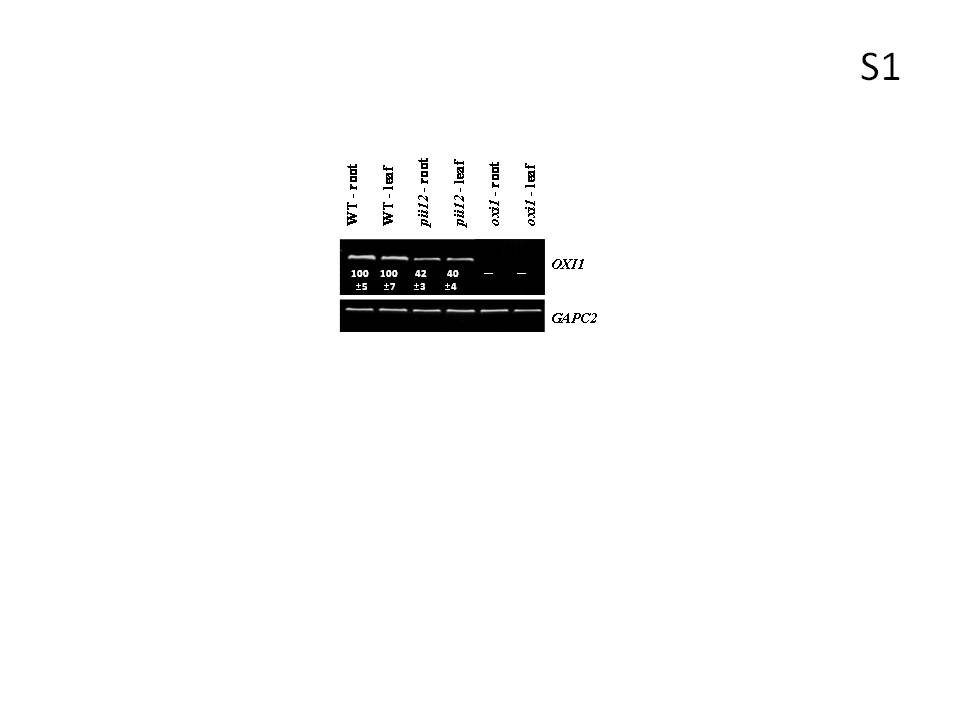
**

1. Visible inspection demonstrated that the root hair length of *pii12* is shorter than that of the wild-type. To obtain quantified data, we determined the average length of the root hair from 200 seedlings, which were grown on MS medium for 10 days on 0.5% phytagel. A picture was taken from root sections of individual seedlings under the microscope and the lengths of all root hairs were measured within an 1.0 mm section (3.0 – 4.0 mm from the root tip).

WT: 379 ± 12 mm

*pii12*: 299 ± 9 mm

*oxi1*: 244 ± 10 mm

*agc2*-2: 391 ± 17 mm.

1. The gene *pii12* was mapped to chromosome 3, using the molecular markers described in Bell and Ecker (1994; Genomics *19*, 137-144). Twenty-three markers for chromosome 3 were taken from <http://www.arabidopsis.org/> servlets/Search;jsessionid=bA0Bx0yKp4iJrq8wZBXQoQ__.tairnode2 to cover most of the chromosome. Ultimately, in a mapping population of 898 individual M3 plants, one recombination event could be detected between the marker SM51_145,1 and no recombination events between the markers GAPA and MDV11I and the mutant locus. GAPA (9.795.222) is located in the vicinity of *oxi1* [31], the only known gene in that region that codes for a signal transduction compound involved in plant/microbe interaction. Furthermore, *pii12* has shorter root hairs, which was also observed for *oxi1* [31]. Sequence analyses of amplified PCR products did not discover any nucleotide differences within the transcribed region of *OXI1.* However, PCR products covering the promoter region of *pii12* lacked 19 nucleotides (TTTTTGCTGCAAGACAAGA) relative to the wild-type.

**Figure S2**

**The *pii12* mutation can be rescued by expression the *OXI1* cDNA under the control of the 35S RNA.**

The picture shows RT-PCR products of *OXI1* mRNA in the roots of *pii12*, wild type (WT), 35S-OXI1-9/*pii12* (#1), 35S-OXI1-11/*pii12* (#2), 35S-OXI1-17/*pii12* (#3), 35S-OXI1-9/wild type (OE) and the *oxi1* knock out line. The construct and transformation was described by [31]. RNA was isolated from the roots of 5 independent plants, grown without the fungus, and the *oxi1* mRNA was quantified by real-time PCR analysis. The *oxi1* mRNA levels are expressed relative to the level of wild-type roots, which was set as 1.0. Growth promotion is based on 3 independent experiments with 10 plants each (cf. Figure 1).


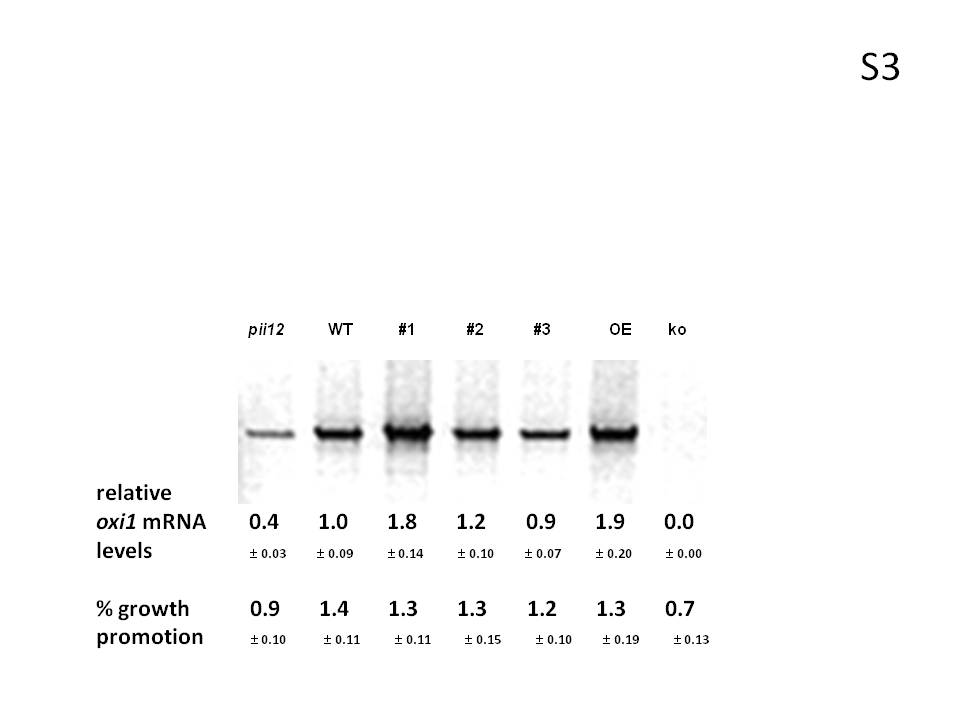


**Figure S3**

**(A) Characterization of *oxi1* and *agc2-2.***

The *oxi1* and *agc2-2* knock out lines do not contain the appropriate transcripts. *OXI1*, *AGC2-2* and *GAPC2* transcript amounts were determined by RT-PCR with gene-specific primer pairs in the roots of 10 day-old seedlings. The *pdk1.1 pdk1.2* mutant is not affected.

**
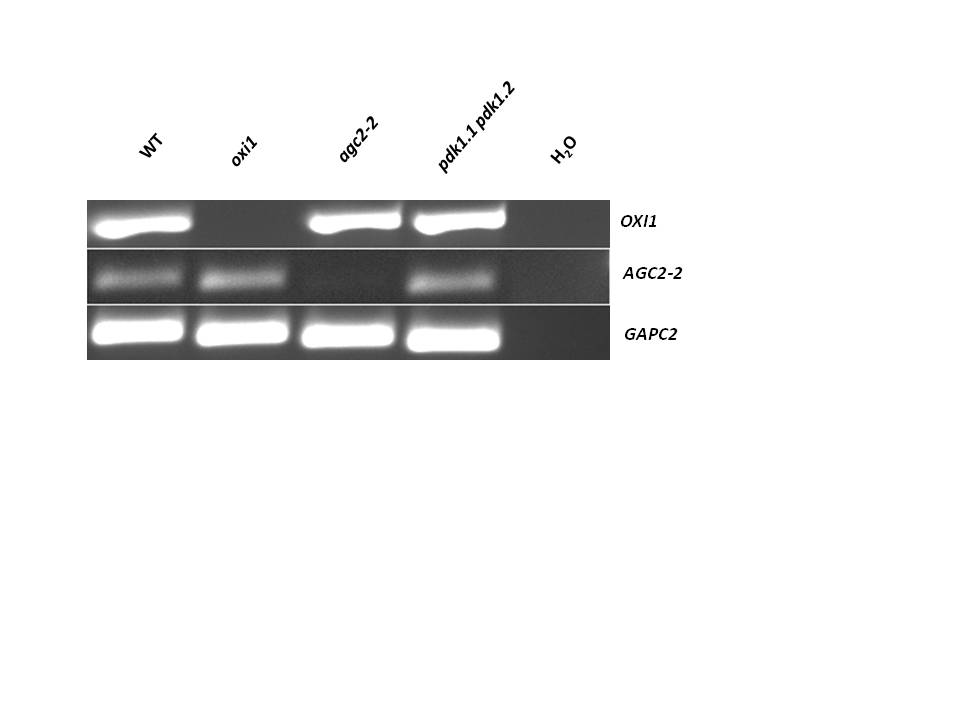
**

**(B) Characterization of *ire.***

The *ire* knock out line does not contain *IRE* transcripts. *IRE* and *GAPC2* transcript amounts were determined by RT-PCR with gene-specific primer pairs in the roots of 10 day-old seedlings. The *oxi1* mutant was used as control. H_2_O, no cDNA was used for PCR.


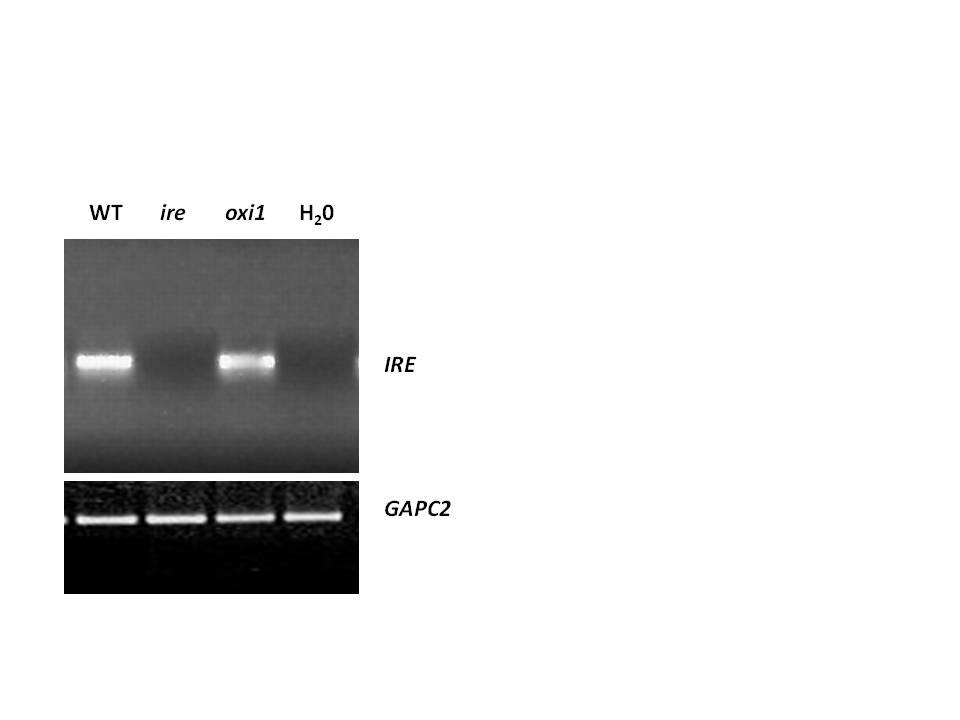


**(C) Characterization of *pldα3* and *pldε.***

The *pldα3* and *pldε* knock out lines do not contain the appropriate transcripts. *PLDα3*, *PLDε* and *GAPC2* transcript amounts were determined by RT-PCR with gene-specific primer pairs in the roots of 10 day-old seedlings. H_2_O, no cDNA was used for PCR.

**Figure S4**

**(A) Arabidopsis seedlings on a fungal lawn.**

Plants grown on a dense fungal lawn. Arabidopsis wild type plants 0 days (beginning of the experiment) and 7 days on a fungal lawn of *P. indica*. Upper parts of the panels: without fungus, lower parts: with fungus. *P. indica* was pre-cultivated on Petri dishes with MS medium for 12 days prior to the beginning of the experiment. The change in the color is caused by the continuation of the fungal growth during the experimental period. NBT stain of the roots. Stain is mainly visible at the lateral root (primordia). No stimulation of H_2_O_2_ is detected on the fungal lawn (cf. also [9]).

**(B) H_2_O_2_ formation on a fungal lawn.**

NBT stain of the roots. Stain is mainly visible at the lateral root (primordia). No stimulation of H_2_O_2_ is detected on the fungal lawn (cf. also [9]).

**(C) H_2_O_2_ formation is repressed in *P. indica*-colonized roots under beneficial co-cultivation conditions.**

Arabidopsis seedlings were co-cultivated (open symbols) or mock-treated (closed symbols) with *P. indica* and the H_2_O_2_ level determined in the roots between 3 and 10 days after infection. Bars represent SEs, based on 9 independent experiments with 30 plants each.


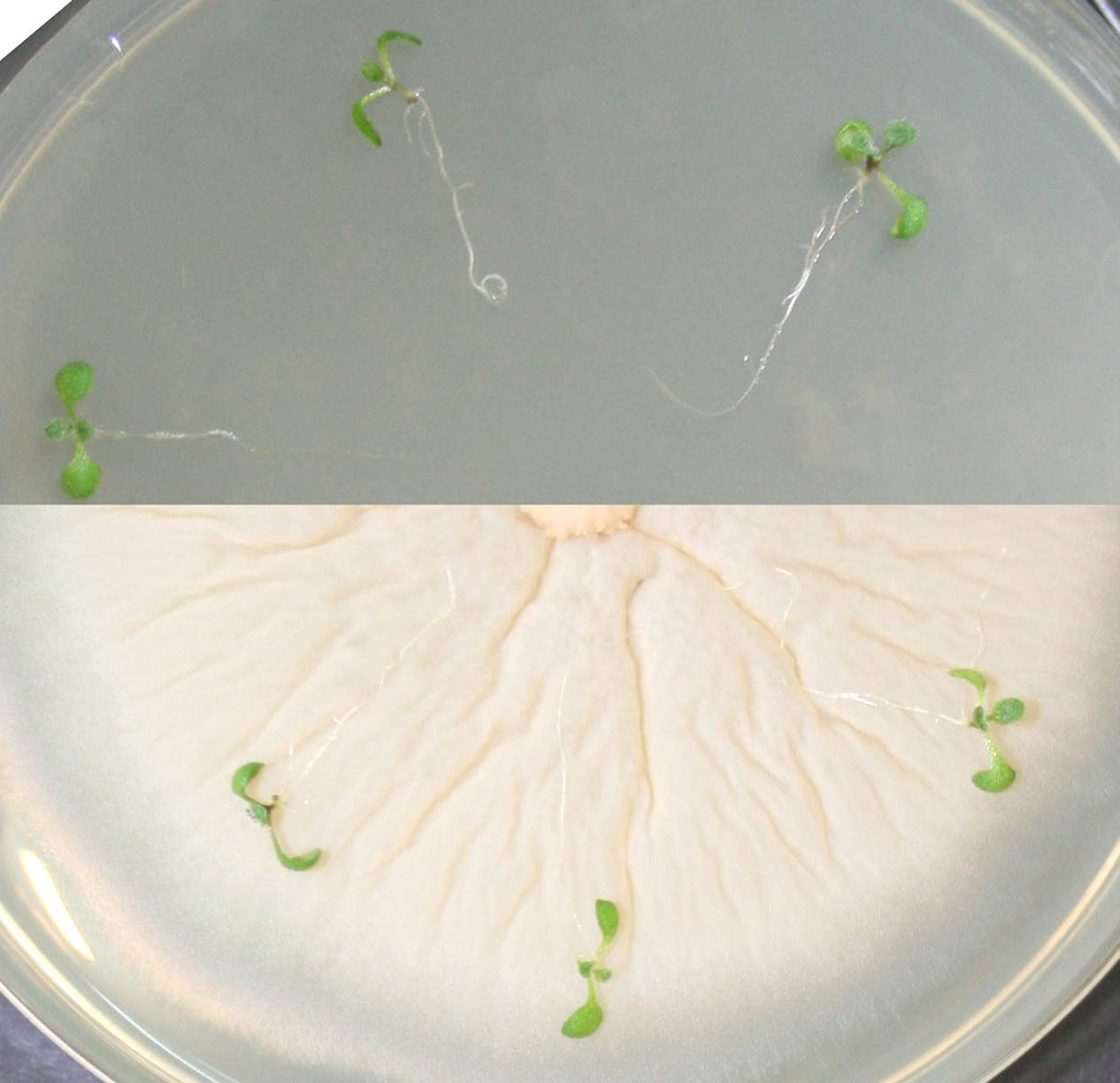

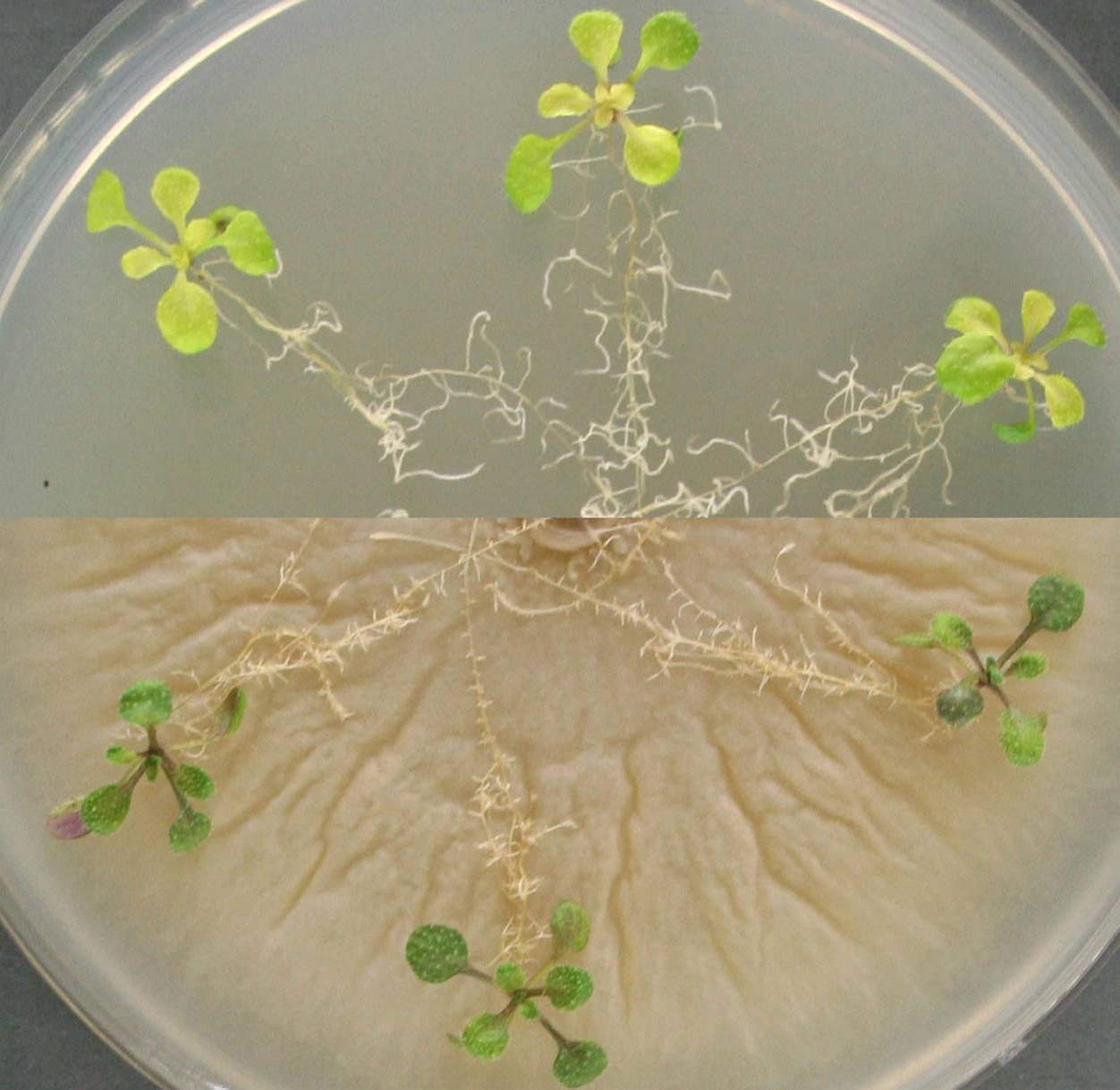


0 dpi

7 dpi

A

B


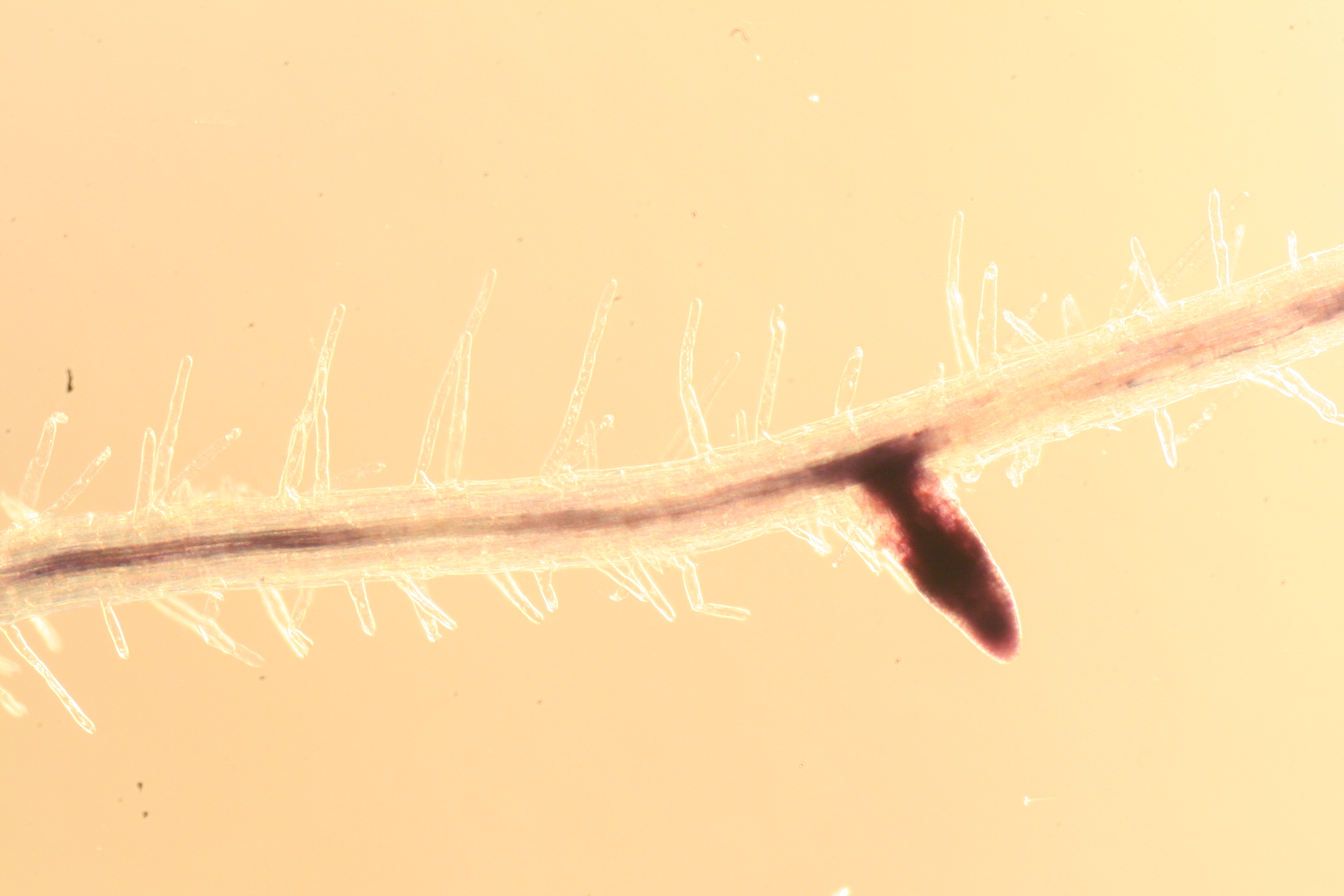

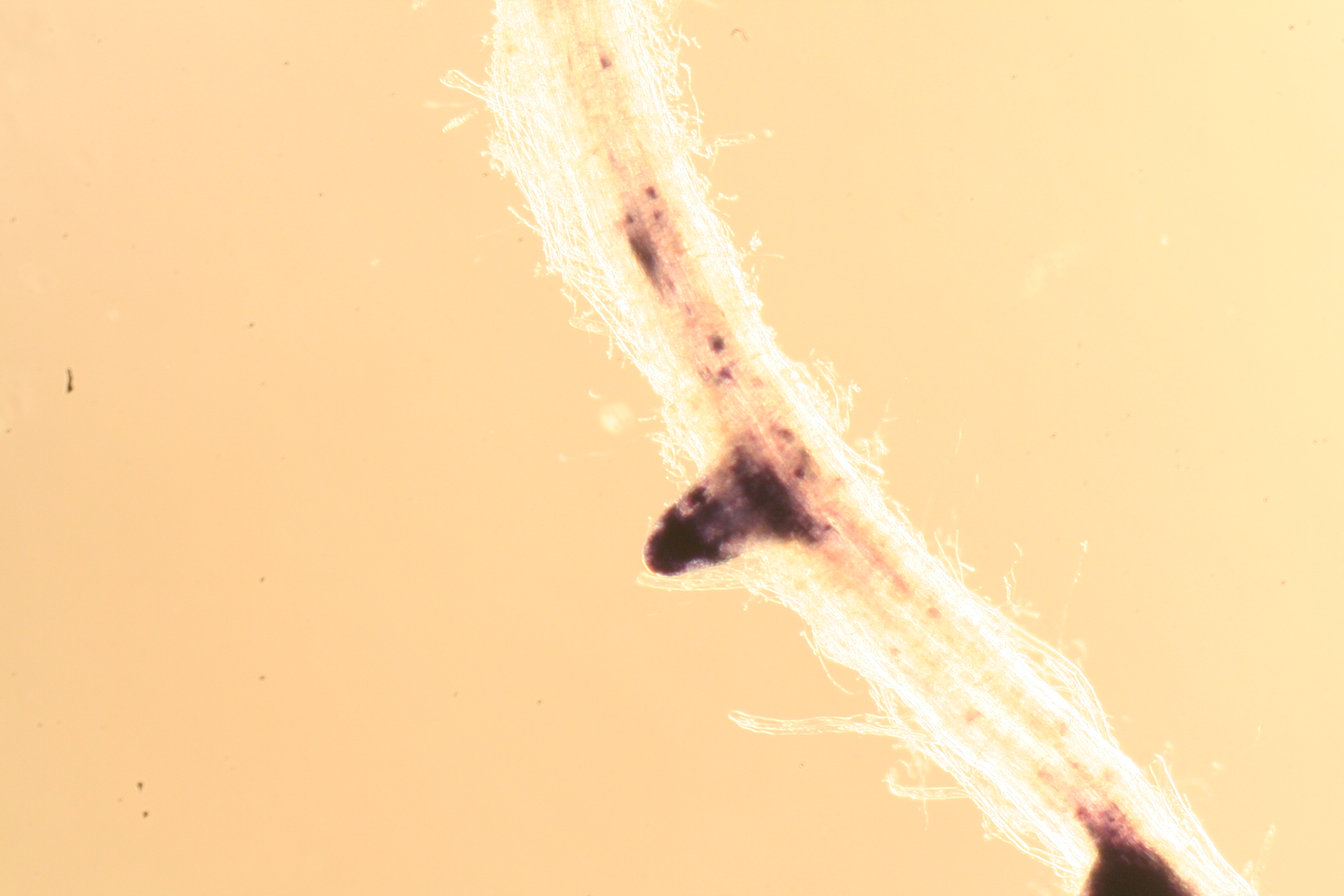


**without fungus**

**fungal lawn**

C

**Figure S5**

Root colonization by *P. indica* is not altered in mutants relative to the wild-type control. Root colonization was calculated as the ratio of the *P. indica* translation elongation factor1 (*Pitef1*) mRNA levels relative to the Arabidopsis *GAPC2* mRNA levels. mRNAs were isolated from Arabidopsis wild-type and mutant roots grown six weeks in the presence of *P. indica* on vermiculite. Based on semiquantitative RT-PCR analysis with 6 independent experiments and 10 plants each, bars represent SEs.

**Figure S6**

Primer pairs used for these studies.

|  | 5´-3´forward | 5´-3´reverse |
| --- | --- | --- |
| *PLDα1-1* (At3g15730) | AGGCTTCCTTGGCAAGATTCT | CCACTTTGGGTTCTTAGGTTC |
| *PLDδ3* (At4g35790) | CAGCATCTGGAATGTGCCAAG | ATTGTCAGCTCCTGCGTCTCT |
| *PDK1.1* (At5g04510) | GTTGTGGATGCTCTTGAGTAT GCTGACTTCAGATGGACACA | TCGTCAGAAGCTGCATTAGG TCTGAAGAGTCTCGATTGCC |
| *PDK1.2* (At3g10540) | AAACTAGCTCCAGATCCTGC CCGGGATTGTGAAACTCTTC | CTGTTGCCATCTTGAATCGAA GAGACTTGAACGTTGAGGTC |
| *OXI1* (At3g25250) | GTGAGTTTCGAGCAAGGAGT AAGAAACGTCTCTTCCGCTTC | GCCGCGTAAAATCTGATAATC TTGATTTCCTTTGCGTTGAAG |
| *AGC2-2* (At4g13000) | GCGACGAAGAAAGAAAGGTCT | **GTTCCAACAAACGAGTTCGAC** |
| *RHD2* (At5g51060) | GACATGGTGGATAAAGATGC | CTCTTCCATGATCAATGCTG |
| *PR-2* (At3g57260) | ACCACACAGCTGGACAAATCG | ATGAGCTCGATGTCAGAGCCA |
| *PR-3* (At3g12500) | TCATGGGGCTACTGTTTCAAG | TATTGCTCTACCGCATAGACC |
| *PDF1-2* (At5g44420) | **CTTGTGTGCTGGGAAGACATA** | AGCACAGAAGTTGTGCGAGAA |
| *ERF1* (At4g17500) | **TATCCTCAACGACGCCTTTC** | **TCTTGACCGGAACAGAATCC** |
| ***PAL2* (At3g53260)** | AGGTACTGACAGTTACGGAG | CATGTCTCCTTCGTGTTTCC |
| *GAPC2* (At3g04120) | GAGCTGACTACGTTGTTGAG | GGAGACAATGTCAAGGTCGG |
| *UBQ5* (At3g62250) | **AAGGCGAAGATCCAAGACAA** | **CGTCGATTCCTTCTGGATGT** |
| *IRE*  (At5g62310) | GCACCAGCTAAGTGATCTTGG | TTTCTACCACAAGGGCATCAG |
| *PLDα3 (At5g25370)* | GGTGGATGAAACAAGGTAGTG | ACCTCCATCCATTGACCTTTG |
| *PLDε (At1g55180)* | ATCAACGGGTTCTTCCCTCT | TCATATCGACCATCGCAAAG |
